# A new data linkage infrastructure.

**DOI:** 10.23889/ijpds.v7i3.2040

**Published:** 2022-08-25

**Authors:** Thomas Alexis

**Affiliations:** 1 Scottish Government

Our overall objectives are to make data linkage a more feasible and frequently used approach by easing the burden on data controllers posed by data assembly for complex linkage projects. By moving towards data linkage as a more expected and standard approach, policy relevant research into a variety of outcomes can be conducted.

The approach to this has been to create a new secure data linkage infrastructure, and to formulate research projects which will use the linked research data delivered by the infrastructure. A suite of information governance documents have also been developed for the data to be held in the secure infrastructure. Holding the data in a de-identified format on behalf of the data controller in a dedicated area for linkage projects means that the data controller only needs to be involved in the approvals process for the project, and a team of experts who operate the infrastructure can assemble the data.

The results have been that there are several datasets pertaining to Education in Scotland being held within the infrastructure, with more in the pipeline. The data is held in such a way that there are no personal identifiers present in the same location as the variables which are typically of research interest; the stored data is split into three parts and shared across two partner organisations. This separation of functions approach assures data controllers and the public that the data poses extremely minimal privacy risk while in storage, but can be assembled into products which are very useful for approved research in the public interest.

In conclusion, the new methodology for data linkage developed by this approach is hoped to save time, money and lives. A dedicated secure environment for data linkage will ensure the security and privacy of stored data while providing capacity for complex, rich research data to be brought together.

